# Short-tailed mice with a long fossil record: the genus *Leggadina* (Rodentia: Muridae) from the Quaternary of Queensland, Australia

**DOI:** 10.7717/peerj.5639

**Published:** 2018-09-21

**Authors:** Jonathan Cramb, Gilbert J. Price, Scott A. Hocknull

**Affiliations:** 1 School of Earth and Environmental Sciences, University of Queensland, Brisbane, Queensland, Australia; 2 Geosciences, Queensland Museum, Brisbane, Queensland, Australia

**Keywords:** *Leggadina*, Muridae, Quaternary, Pleistocene, Rodents, Palaeoecology, Extinction, Climate change

## Abstract

The genus *Leggadina* (colloquially known as ‘short-tailed mice’) is a common component of Quaternary faunas of northeastern Australia. They represent a member of the Australian old endemic murid radiation that arrived on the continent sometime during the late Cenozoic. Here we describe two new species of extinct *Leggadina* from Quaternary cave deposits as well as additional material of the extinct *Leggadina macrodonta*. *Leggadina irvini* sp. nov. recovered from Middle-Upper (late) Pleistocene cave deposits near Chillagoe, northeastern Queensland, is the biggest member of the genus, being substantially larger than any other species so far described. *Leggadina webbi* sp. nov. from Middle Pleistocene cave deposits at Mount Etna, central eastern Queensland, shares features with the oldest species of the genus, the Early Pleistocene *L. gregoriensis*. Based on the current palaeoecological interpretation of the type locality, *L. webbi*, represents the only member of the genus that inhabited rainforest. The succession of *Leggadina* species through the late Quaternary suggests an ecological replacement of the extinct large-bodied *L. irvini* with the extant, small-bodied *L. lakedownesis* at Chillagoe. At Mt. Etna, the extinct rainforest species *L. webbi* is replaced with the extant xeric-adapted *L. forresti* during the latest Middle Pleistocene. This replacement is associated with a mid-Pleistocene shift towards progressive intensifying seasonal and arid climates. Our study adds to the growing list of small-bodied faunal extinctions during the late Quaternary of northern Australia.

## Introduction

Short-tailed mice (*Leggadina* spp.) are small-bodied murines that form part of the ‘Australian Old Endemic Radiation’ (Aplin, 2006; Watts & Aslin, 1981), now referred to the tribe Hydromyini (Lecompte et al., 2008). The genus has a complex taxonomic history. Thomas (1906) was the first to describe a species of *Leggadina*, but placed it in the genus *Mus*. Thomas later proposed the name *Leggadina* as a subgenus of *Pseudomys* (Thomas, 1910). Iredale & Troughton (1934) elevated *Leggadina* to full generic status, a move supported by Ellerman (1941) and Tate (1951). Several early workers (Thomas, 1910; Ellerman, 1941; Tate, 1951) placed any very small Australian rodent species with an accessory cusp on M^1^ in *Leggadina*, but some authors (Ellerman, 1941; Tate, 1951) noted that several species (now synonymised into *L. forresti*) were distinct from all others. Ride (1970) synonymised *Leggadina* back into *Pseudomys*, only to have it resurrected again by Mahoney & Posamentier (1975).

Covacevich & Easton (1974) considered that the Central Short-tailed Mouse, *L. forresti* (listed as *Pseudomys forresti*), was widespread across Queensland. However, individuals from the northeast of Queensland were later shown to be distinct by Watts (1976) and Baverstock et al. (1976), and subsequently named *Leggadina lakedownensis*. Later authors considered that all *Leggadina* in northern Australia were conspecific with *L. lakedownensis* (Moro et al., 1998), with *L. forresti* restricted to the continent’s interior. No additional extant species of *Leggadina* have been described since that time, although regional variants have been recognised (Cooper et al., 2003).

*Leggadina* is traditionally included in the ‘*Pseudomys* division’ (‘Conilurini’), which also includes *Conilurus*, *Mesembriomys*, *Leporillus*, *Notomys*, *Zyzomys*, *Mastacomys* and *Pseudomys* itself (Musser & Carleton, 2005). However, molecular phylogenies indicate that *Conilurus*, *Mesembriomys* and *Leporillus* are actually the sister group of the ‘*Uromys* division’ (*Uromys*, *Melomys*, and related genera), making the ‘*Pseudomys* division’ paraphyletic (Rowe et al., 2008; Steppan et al., 2005). Regardless, the position of *Leggadina* as a close relative of *Zyzomys* and a *Pseudomys*/*Notomys*/*Mastacomys* clade is well-supported (Ford, 2006; Rowe et al., 2008). Most recently an analysis by Smissen & Rowe (2018) found *Leggadina* to be the sister clade of *Zyzomys*.

*Leggadina* has a relatively long fossil record for an Australian murine genus spanning much of the Quaternary. The earliest records come from the Early Pleistocene Riversleigh World Heritage Area, northwestern Queensland (Klinkhamer & Godthelp, 2015) and Fisherman’s Cliff fossil deposits in southern New South Wales (Crabb, 1977; Breed & Ford, 2007). Middle Pleistocene reports include Hocknull (2005; 2009) and Hocknull et al. (2007) from cave deposits at Mt. Etna, central Queensland. Recent owl roost deposits in central Australia commonly contain the skeletal and dental remains of *L. forresti* (e.g. Baynes & Johnson, 1996), whilst *L. lakedownensis* has been recorded from cave deposits in northern Western Australia (Start et al., 2012).

In light of new material now available for analysis, we here review the fossil record of *Leggadina* in northeastern Australia, erect two new species from fossils of Pleistocene age, and describe additional material referrable to the extinct *L. macrodonta*. The evolution and palaeoecology of species of *Leggadina* are discussed, with implications for our understanding of the wider Australian murine fauna.

## Material and Methods

**Figure 1:**
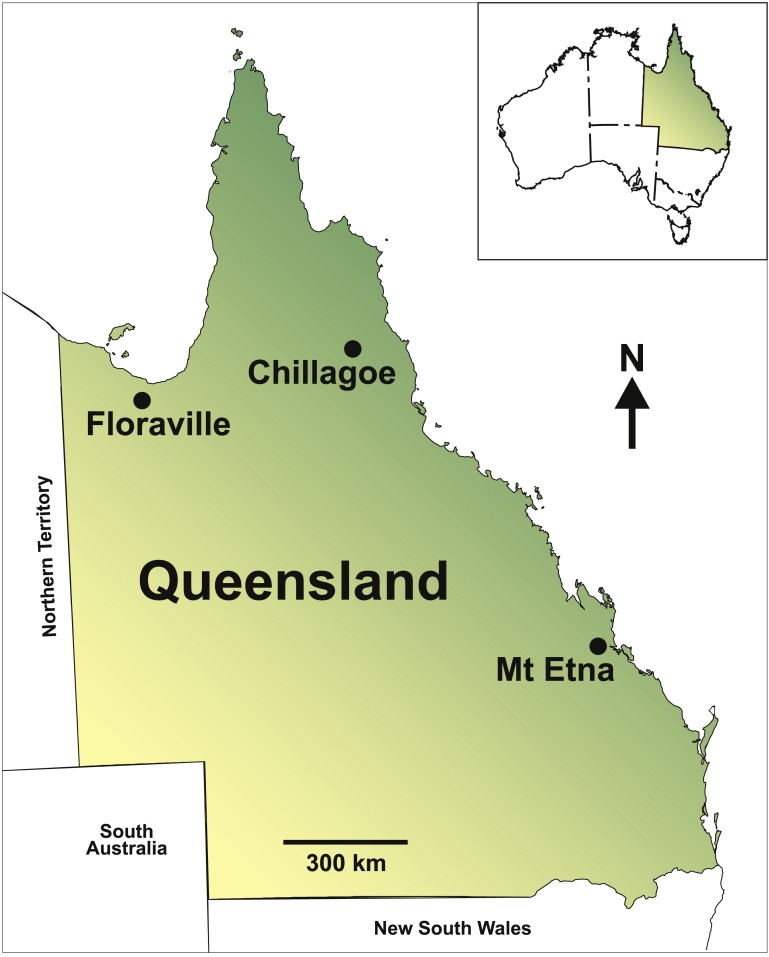
Map of Queensland, Australia, showing the study localities.

Specimens were obtained as part of ongoing palaeontological investigations of Pleistocene deposits in Queensland. These include the fossil-rich cave deposits of the Mount Etna (central eastern Queensland) and Chillagoe (northeastern Queensland) areas, and open fluvial deposits at Floraville (northwestern Queensland) (Fig. 1). The fossil material comprises maxillae, dentaries and isolated teeth. All fossils described in this paper are accessioned into the collections of the Queensland Museum, Brisbane, Australia.

**Figure 2:**
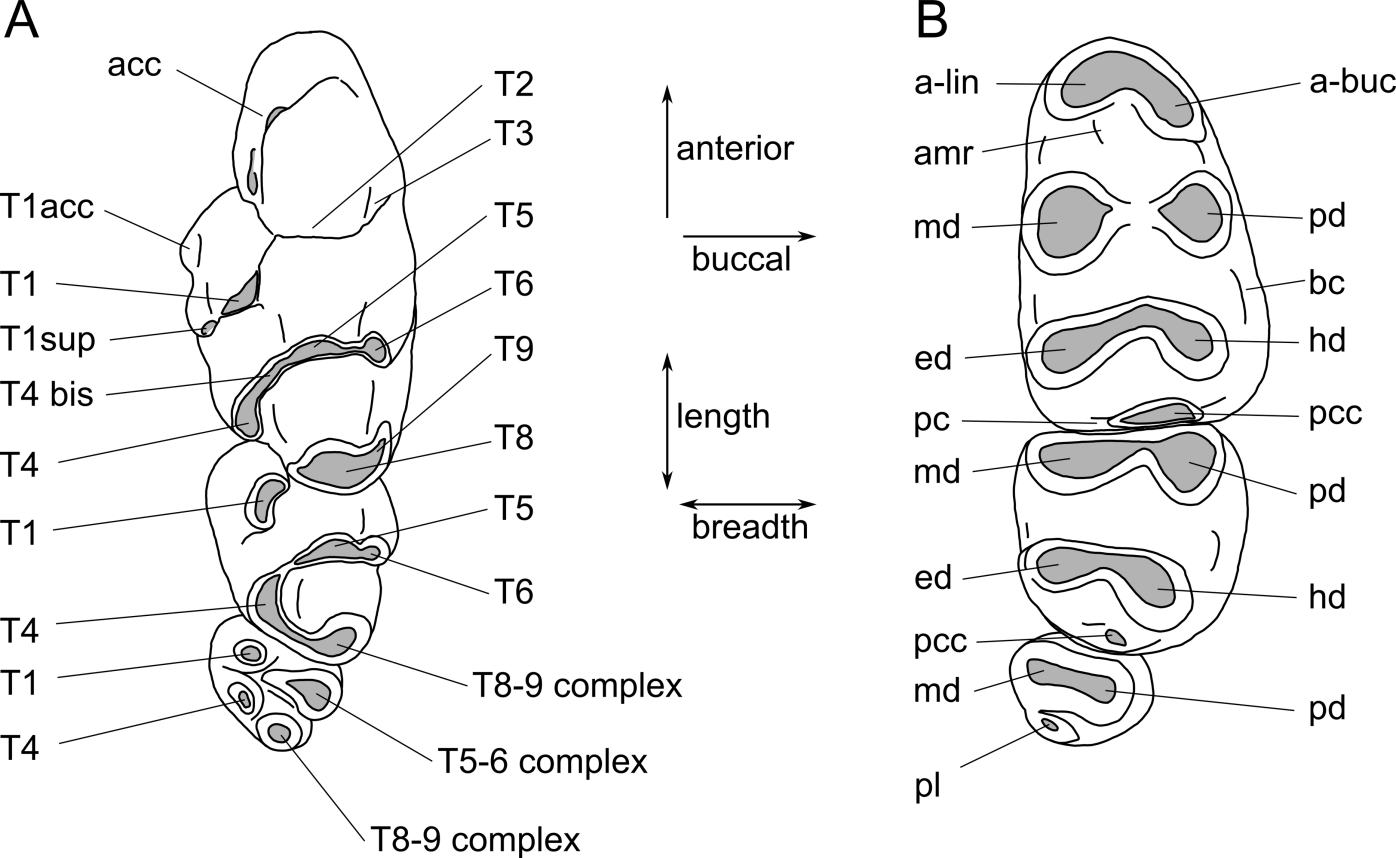
Molar cusp terminology for species of *Leggadina*. (A) Upper molars. (B) Lowers molars. Terminology is modified from Musser (1981) and Aplin & Helgen (2010). Abbreviations: acc, accessory cusp; T1acc, T1 accessory cusp; T1sup, T1 supplementary cusp; a-lin, antero-lingual cuspid; a-buc, antero-buccal cuspid; amr, anterior medial ridge; md, metaconid; pd, protoconid; ed, entoconid; hd, hypoconid; bc, buccal cingulid; pc, posterior cingulid; pcc, posterior cingulid cuspid; pl, posterior loph. The term ‘complex’ is used where individual cusps cannot be distinguished.

Images were obtained using a Visionary Digital ‘passport storm’ camera system and a Hitachi TM-1000 environmental scanning electron microscope at the Queensland Museum. Measurements were taken using Mitutoyo digital callipers. Dental terminology follows Fig. 2.

The electronic version of this article in Portable Document Format (PDF) will represent a published work according to the International Commission on Zoological Nomenclature (ICZN), and hence the new names contained in the electronic version are effectively published under that Code from the electronic edition alone. This published work and the nomenclatural acts it contains have been registered in ZooBank, the online registration system for the ICZN. The ZooBank LSIDs (Life Science Identifiers) can be resolved and the associated information viewed through any standard web browser by appending the LSID to the prefix http://zoobank.org/. The LSID for this publication is: urn:lsid:zoobank.org:pub:3C34C73B-4800-4256-BC9E-0E7B50689024. The online version of this work is archived and available from the following digital repositories: PeerJ, PubMed Central and CLOCKSS.

### Geographic and geological settings

#### Floraville

Numerous vertebrate-bearing fossil deposits occur in the vicinity of Floraville Downs Station in north-western Queensland (Fig. 1). The deposits are predominantly fluviatile, occurring adjacent to the western banks of the Leichhardt River and associated tributaries. The fossil-bearing sediments consist of silts, clays, sands, and gravels, including some with extensive *in situ* carbonates.

Fossil collecting on the station has been sporadic since the 1970s, with no single deposit subjected to extensive and detailed excavation. The majority of finds have been of surface specimens and include mostly megafaunal taxa (i.e., large-bodied extinct Quaternary vertebrates including giant marsupials and crocodiles). Where fossil collecting has adopted techniques conducive to the recovery of small-bodied species (e.g., sieving of sediments and fossils using fine-mesh sieves from selected deposits), significant and diverse vertebrate-rich assemblages have been recovered (Rich, 1991). The deposits have previously been considered to be Plio-Pleistocene (Godthelp, 1999; Tyler, Archer & Godthelp, 1994), however, recent radiometric dating strongly suggests that the deposits are Middle Pleistocene (Price, 2013). Moreover, the fossil assemblages lack typical Pliocene taxa such as the large-bodied wombat-like marsupial *Euryzygoma*, but have yielded numerous specimens of the closely related *Diprotodon*, a species found exclusively in Pleistocene deposits ( Price et al., 2009).

#### Chillagoe

Fossil assemblages from the Chillagoe area, north-eastern Queensland (Fig. 1) have been recovered from cavernous and fissure-fill deposits within outcrops of Devonian karst limestone (Price, 2013; Price et al., 2013). The new records of *Leggadina* from Chillagoe are derived from two cave systems, Macropedes Waterhole Cave and Fern Cave.

The Macropedes Waterhole Cave deposit is currently undated, but contains both extant species and multiple extinct taxa, including hitherto undescribed murine rodents (Cramb, 2012). The fossil assemblage also contains specimens assignable to *Rattus* (Cramb, 2012), a genus that is purported to have arrived in Australia around 0.85–1.28 Ma (Rowe et al., 2011), thus potentially constraining an upper age limit for the deposit as Early Pleistocene. The brecciated deposit is dominated by clays with occasional bone clasts. The sediments within the deposit clearly have a long geological history having accumulated within the cave, lithified, and then subsequently eroded. We, therefore, hypothesise that the deposit is Middle to Late Pleistocene in age.

In 2009 during an exploratory search of the cave for fossils, a small (∼125 g) sample was collected from a hollow in the breccia, where eroded fossils had accumulated. After the significance of the sample was ascertained it was hoped that additional fieldwork would recover additional samples. Unfortunately the cave was flooded at the time of a subsequent field trip, making the deposit inaccessible.

Previous investigations at Fern Cave have targeted excavation of an archaeological deposit within it (e.g., Goodall et al., 2009). However, *Leggadina* specimens examined in this paper were derived from an active owl-roost positioned on a ledge high above the deposit. The specimens were collected at the time of the original archaeological investigations (ca. 1985 AD), but have remained unpublished until now. The sediments are unconsolidated, and were excavated in five cm spits. The owl roost has not been dated, but at least some of the skeletal material within it is clearly Recent. Based on the size of the deposit we suspect the fauna are Holocene to recent in age.

#### Mt. Etna region

Fossil specimens of *Leggadina* collected from the Mt. Etna region have come from numerous limestone cave deposits, located on Mt. Etna, the adjacent Limestone Ridge and nearby Olsen’s Cave (Hocknull, 2005; Hocknull et al., 2007; Hocknull, 2009; Price & Piper, 2009; Cramb, Hocknull & Webb, 2009; Cramb & Hocknull, 2010a; Price et al., 2015). The majority of the fossil-rich deposits at Mt. Etna were initially exposed as a result of limestone mining activities on the western side of Mt. Etna, while fossil deposits at Limestone Ridge and Olsen’s Cave are derived from naturally exposed deposits or deposits exposed through guano mining. The fossil deposits are variable in their lithology, ranging from phreatically-derived brecciated, red clay-dominated sediments through vadose-derived, speleothem-rich yellow clay-rich sediments to guano-derived, chocolate-brown organic-rich sediments (Hocknull, 2009). All deposits are variably cemented with carbonate; well-cemented samples were collected as blocks and digested in acetic acid baths. Less consolidated deposits were excavated in five cm spits where possible. One important site (QML1312 Elephant hole Cave) was destroyed in 1988–1989, before systematic sampling could be undertaken. It is nevertheless well represented by a sample collected by cavers prior to the caves’ destruction (Hocknull, 2005).

Uranium-thorium dating places the majority of deposits in the area within the last 500 ka, with some (e.g., Olsen’s Cave) as recent as the late Holocene (Hocknull et al., 2007; Price et al., 2015). The older deposits in the area (>500–280 ka BP) contain numerous species interpreted as indicative of a closed rainforest palaeoenvironment, while deposits dated to <250 ka BP mostly contain taxa that are more indicative of open, xeric habitats (Hocknull, 2005; Hocknull et al., 2007).

## Results

### Systematic palaeontology

**Table:** 

Class Mammalia Linnaeus, 1758
Subclass Theria Parker and Haswell, 1897
Supercohort Placentalia Bonaparte, 1838
Order Rodentia Bowdich, 1821
Family Muridae Illiger, 1811
*Leggadina*Thomas, 1910

**Table:** 

Type species: *Mus forresti*Thomas, 1906
Other species: *L. lakedownensis*Watts, 1976
*L. gregoriensis*Klinkhamer & Godthelp, 2015
*L. macrodonta*Klinkhamer & Godthelp, 2015


Generic diagnosis. Species of *Leggadina* are distinguished from all other murids by a unique combination of characters including the possession of: large accessory cusp on M^1^; large, lingual cusps on M^1−2^ postero-lingual of associated lophs; commonly diamond-shaped T1 on M^1^; T1 supplementary cusp commonly present on M^1^, although variably expressed; upper molar cusps sloped posteriorly; M^1^/_1_ enlarged; M^3^/_3_ reduced, such that M^1^/_1_ is longer than M^2−3^∕_2−3_ combined; large posterior palatal foramen; broad posterior cingulid cuspid on M_1_; lower molar cusps sloped anteriorly; apex of each lower molar cusp drawn in from margins of base.

Generic remarks.

*Leggadina* can be separated from all other Australo-Papuan murines by the large accessory cusp on M^1^, large posterior palatal foramen, enlargement of M^1^/_1_ with associated reduction of M^3^/_3_ so that M^1^/_1_ is more than half the length of the molar row. Small species of *Pseudomys* (e.g., *P. delicatulus*) share some similarities with *Leggadina*, but differ by having a smaller accessory cusp on M^1^, less development of the T1 supplementary cusp on M^1^, smaller posterior palatal foramen, proportionally smaller M^1^/_1_ and larger M^3^/_3_ (M^1^/_1_ commonly half the length of the molar row), and a smaller posterior cingulid cusp on M_1_.

Morphological differences between the two extant species of *Leggadina* (see Watts, 1976; Cooper et al., 2003; Van Dyck, Gynther & Baker, 2013) are partially based on intact skulls. However, most fossils are represented by fragmentary remains, such that only a subset of features can be used to diagnose fossil species within *Leggadina*. Those characters include: the length and width of M^1^; length of accessory cusp on M^1^; relative length of M^2^ compared to M^1^; relative reduction of M^3^; length of M_1_; and the shape of the posterior end of the anterior palatal foramen.

**Figure 3:**
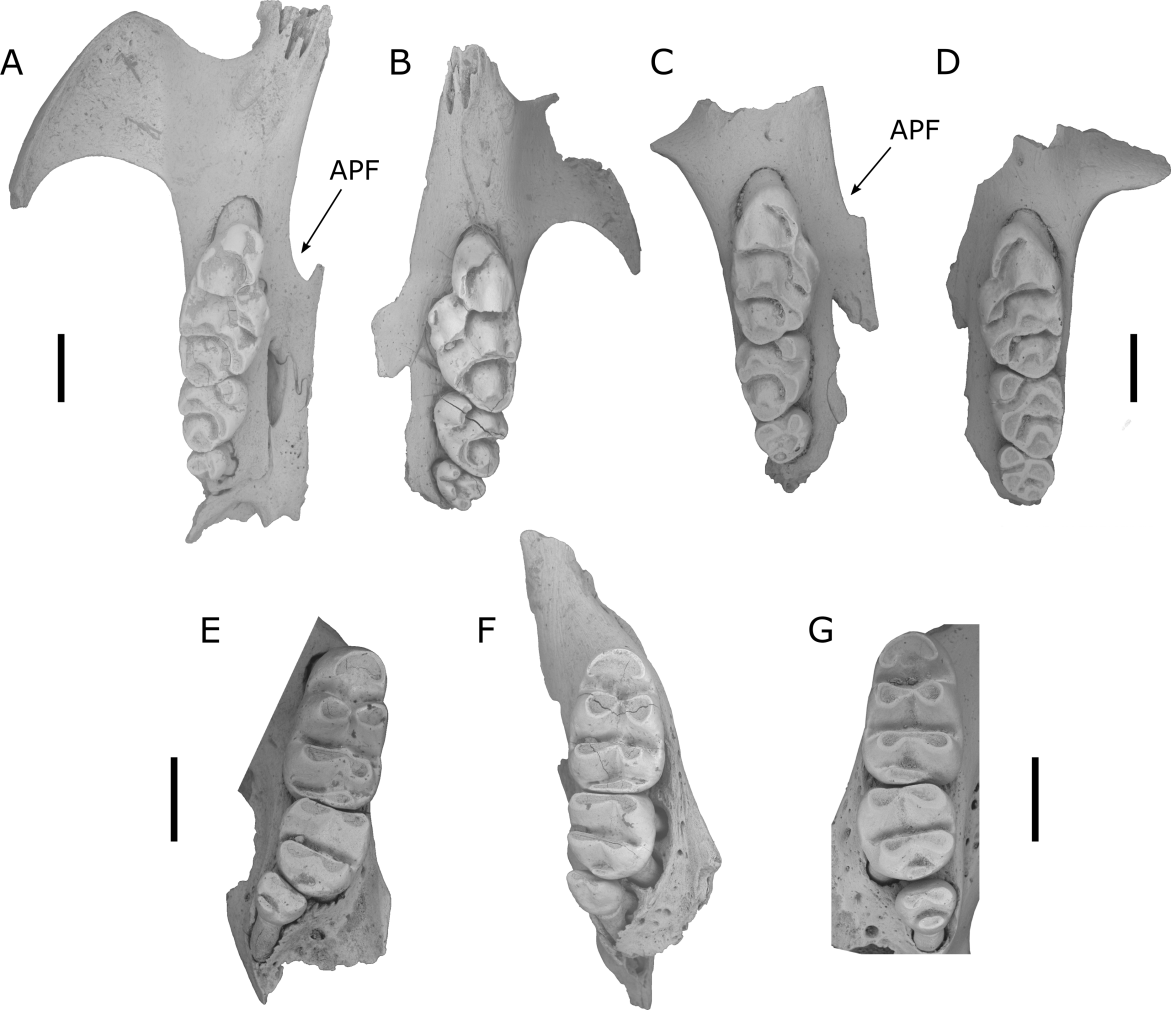
Fossils of *L. lakedownensis* and *L. forresti* from cave deposits at Chillagoe and Mount Etna. (A–D) Upper dentions: (A) QMF55619 *L. lakedownensis* right maxilla; (B) *L. lakedownensis* QMF55618 left maxilla; (C) QMF58982 *L. forresti* right maxilla; (D) QMF58983 *L. forresti* left maxilla. (E–G) Lower dentitions: (E) QMF55621 *L. lakedownensis* right mandible; (F) QMF55620 *L. lakedownensis* right mandible; (G) QMF58981 *L. forresti* left mandible. Scale bar = 1 mm. APF, anterior palatal foramen. The buccal edge of the APF is curved in *L. lakedownensis*, but straighter in *L. forresti*.

***Leggadina forresti*** (Thomas, 1906) (Fig. 3)

Diagnosis: See Thomas (1906), to which we can add the following features that have proved useful when identifying fossil specimens: *Leggadina forresti* is a small species within the genus and possesses the following unique combination of characters: enlarged M^1^/_1_, heavily reduced M^3^/_3_, strongly developed accessory cusp on M^1^ (variably divided), moderately to well-developed T1 supplementary cusp on M^1^, and an anterior palatal foramen that has a straight buccal edge on the maxilla and tapers to a point posteriorly.

Material. QML1312; Elephant Hole Cave System, Mount Etna: QMF55341 left maxilla, QMF55646 left maxilla, QMF55647 right mandible, QMF55648 left M^1^; QML1313A; Speaking Tube Cave System subsample A, Mount Etna: QMF55649 left maxilla fragment with M^1^, QMF55649 left maxilla fragment with M^1^; QML1456; Colosseum Chamber, Capricorn Caves: QMF55148 left maxilla, QMF55149 right maxilla, QMF55150 right maxilla, QMF55151 right maxilla, QMF55152 left mandible, QMF55153 right mandible, QMF55767 right maxilla, QMF58981 left mandible; QML1466 Honeymoon Suite, Capricorn Caves: QMF58982 right maxilla, QMF58983 left maxilla.

Remarks.

*L. forresti* is identified here by its large accessory cusp (larger than those of *L. gregoriensis* and *L. webbi*), relatively small size (dentition smaller than *L. macrodonta* and *L. irvini* sp. nov.), and relatively straight buccal margin of the anterior palatal foramena, which also tapers to a point posteriorly (unlike the curved buccal edge and commonly rounded posterior termination of the anterior palatal foramena in *L. lakedownensis*). *L. forresti* occurs in several Middle Pleistocene to Holocene cave deposits in the Mount Etna region, with the oldest record from a subsample of Speaking Tube Cave (designated QML1313A) with a minimum age of 280 ka. Interestingly, while the main QML1313 assemblage is dominated by hydric-adapted fauna interpreted as a rainforest palaeoecology at the time of deposition (Hocknull, 2005; Hocknull, 2009; Hocknull et al., 2007), the QML1313A sample contains a peculiar mixture of hydric and xeric-adapted taxa. This is in stark contrast to the known habitat of extant *L. forresti*, a species found in grasslands, shrublands and sparsely vegetated plains of inland Australia (Reid, 2008).

***Leggadina lakedownensis*** Watts, 1976 (Fig. 3)

Diagnosis: See Watts (1976).

Material: QML1466; Fern Cave - owl roost deposit: QMF55269 partial skull, QMF55353 right maxilla, QMF55354 left maxilla, QMF55355 right maxilla, QMF55356 left M^1^, QMF55618 left maxilla, QMF55619 right maxilla, QMF55620 right mandible, QMF55621 right mandible.

Remarks.

The above specimens are referred to *L. lakedownensis* because they possess an enlarged M^1^/_1_, heavily reduced M^3^/_3_, strongly developed accessory cusp on M^1^ (commonly divided), prominent T1 supplementary cusp, and an anterior palatal foramen that is commonly rounded posteriorly. Of these characters, the shape of the anterior palatal foramina seems to be the most useful character for distinguishing specimens of *L. lakedownensis* from *L. forresti* in Queensland, although Cooper et al. (2003) found it to be variable and therefore less useful in specimens from Western Australia. Watts (1976) stated that the posterior end of the anterior palatal foramen is rounded in *L. lakedownensis*. This is certainly the case in the holotype and two of the paratypes, but one paratype (QMJM1293) has it pointed instead. We suggest that a more useful characterisation of this feature is that the buccal margin of the anterior palatal foramen of *L. lakedownensis* is curved, while that of *L. forresti* is relatively straight.

Specimens of *L. lakedownensis* have thus far been recorded only within the Recent owl roost of Fern Cave in the Chillagoe area, but do not occur within the older fossil breccias.

**Figure 4:**
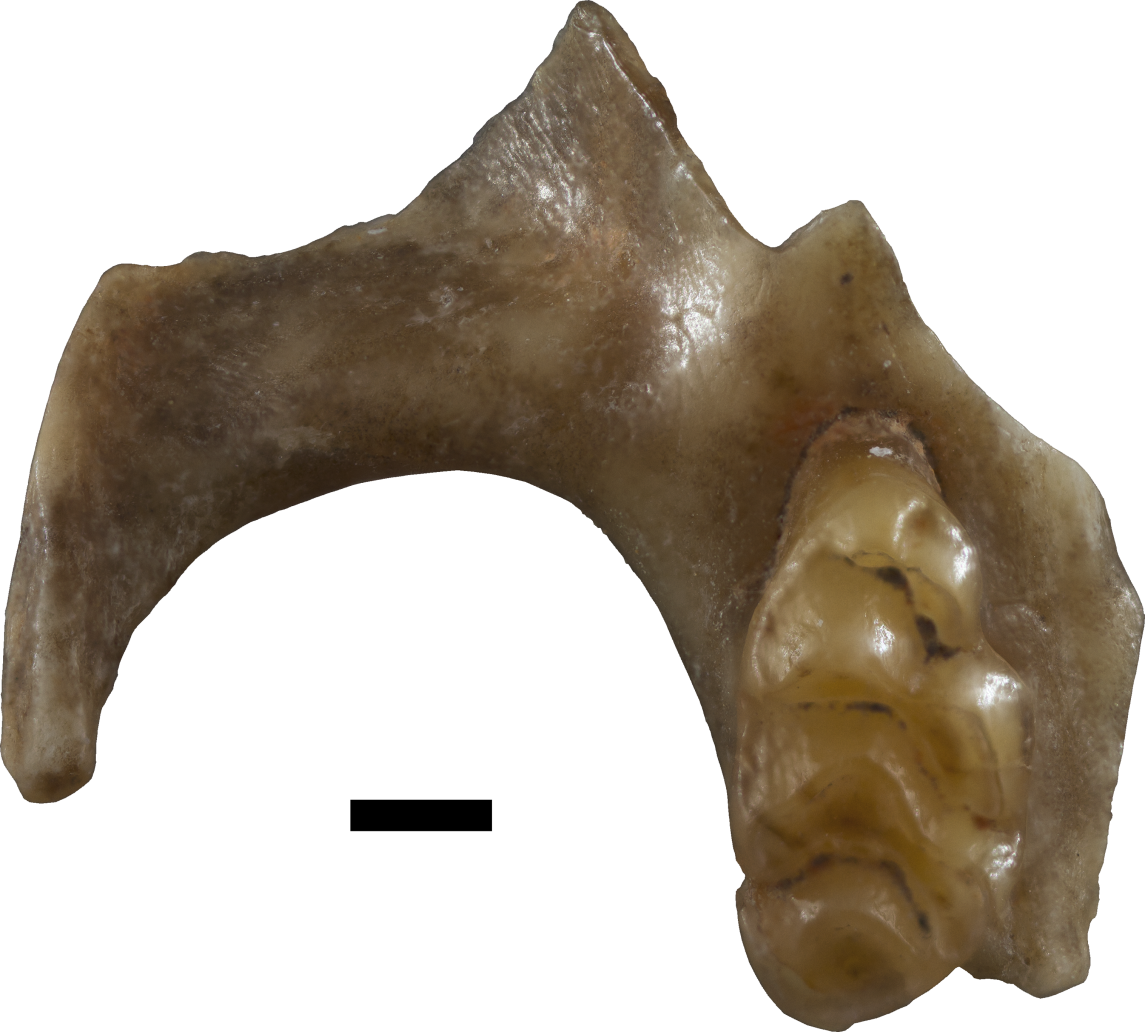
*Leggadina. irvini* sp. nov. QMF55326 right maxilla fragment with M^1^ from QML1507 Macropedies Waterhole breccia. Scale bar = 1 mm.

**Figure 5:**
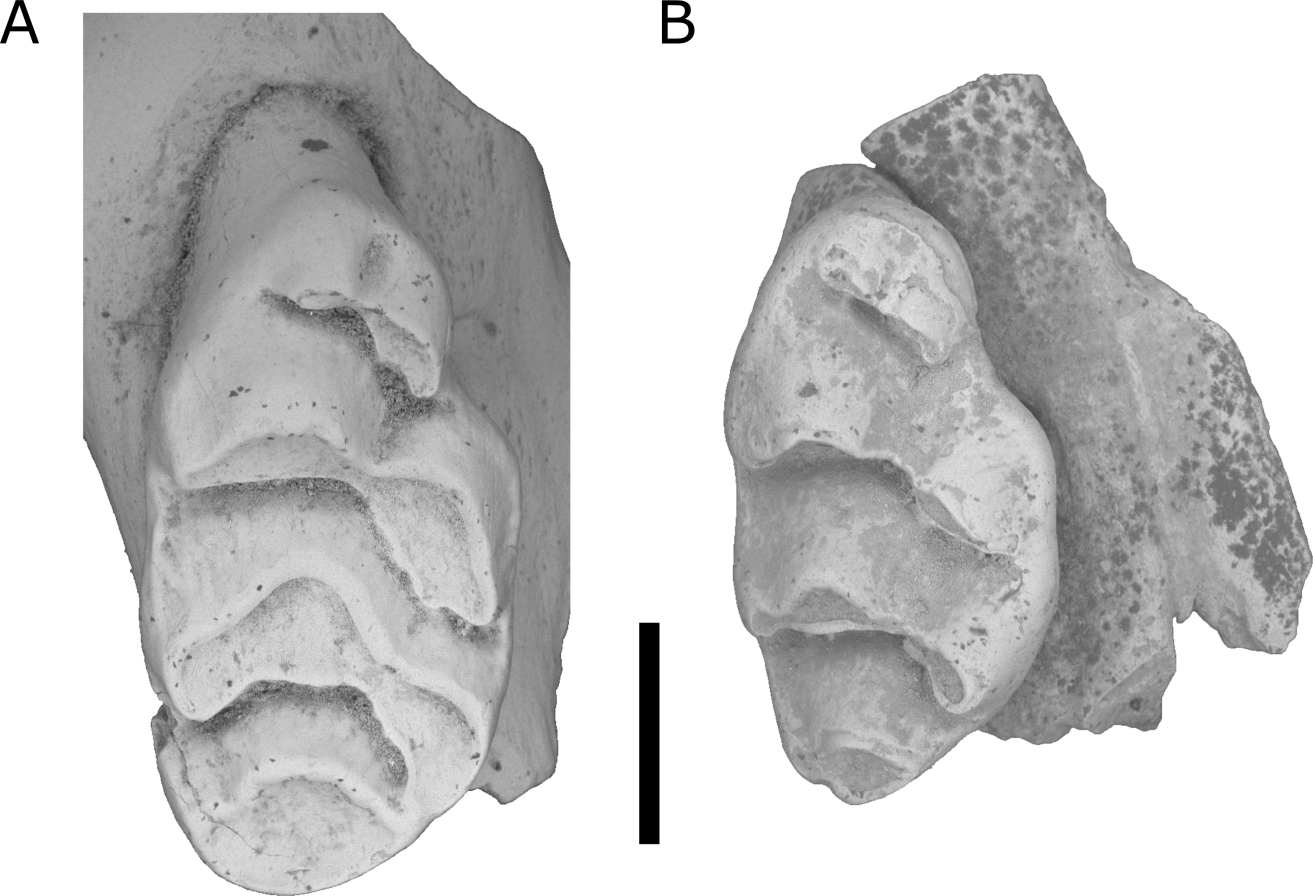
Comparison of right M^1^ of *L. irvini* with *L. macrodonta*. (A) QMF55326 *L. irvini*. (B) QMF55624 *L. macrodonta* right M^1^. Scale bar = 1 mm.

***Leggadina*** ***irvini*** **sp. nov.** (Figs. 4–5)

Holotype. QMF55326 right maxilla fragment with M^1^.

Type locality. QML1507; Macropedies Waterhole Cave breccia, Chillagoe, north-eastern Queensland. A massive breccia that once filled much of the chamber, since eroded to relatively small blocks that are still *in situ*. The age of the breccia is uncertain, but is likely Middle or Late Pleistocene.

Etymology: This species is named for Mr. Douglas Irvin in honour of his contributions to, and support of, palaeontological work in caves of eastern Australia.

**Figure 6:**
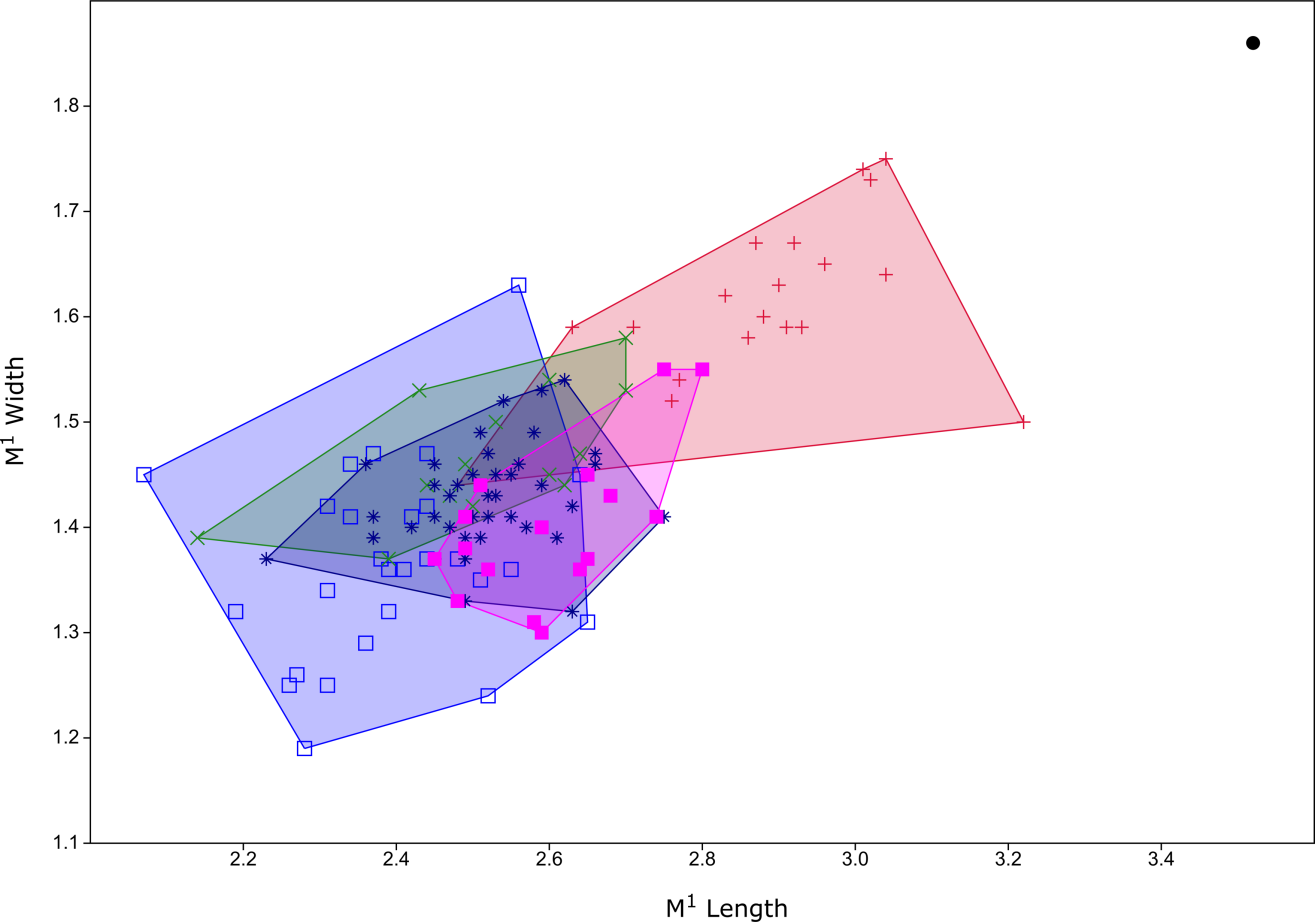
Plot of M^1^ dimensions for species of *Leggadina* from Quaternary fossil deposits in Queensland. Blue open square = *Leggadina gregoriensis* (Rackham’s Roost); green X = *Leggadina webbi* (QML1311H,1311J, 1313); dark blue asterisk, *Leggadina forresti* (QML1312, 1456, 1457); purple filled square, *Leggadina lakedownensis* (QML1466); red +, *Leggadina macrodonta* (Floraville 5C); black dot, *Leggadina irvini* (QML1507). Measurements in millimetres.

Diagnosis. A species of *Leggadina* that differs from all other members of the genus by being substantially larger than any other currently known species (Fig. 6). Additionally, the M^1^ possesses a well-developed accessory cusp (divided in holotype and only known specimen), and large diamond-shaped T1 in occlusal outline.

Description.

Maxilla. QMF55326 preserves the portion of the maxilla surrounding M^1^ and part of the zygomatic arch; broken edges of maxilla slightly rounded and polished due to erosion from breccia; shape and position of the anterior palatal foramen cannot be discerned with certainty; portion of anterior margin of posterior palatal foramen may be preserved, but damage and rounding of specimen makes this uncertain.

M^1^. Crown elongate, oval-shaped in occlusal outline with a marked antero-lingual indent; Cusps moderately worn, but appear to be relatively low-crowned; accessory cusp very large, divided into two (smaller, oval-shaped anterior cusplet; larger crescent-shaped lingual cusplet); T1 shaped like a diamond in occlusal outline; T1 large, robust, slightly smaller than T2-3 complex; T1 postero-lingual of T2; T3 in-line with T2; T2 posterior edge forms a 116 degree angle with T1; T3 heavily reduced to a small, buccal projection of T2; T4-6 loph more worn than T1-3; T4 similar in size to T1 but semicircular-shaped in occlusal outline; T5 antero-buccal of T4 and antero-lingual of T6; T6 much larger than T3, possibly more distinct when unworn; T4 joined to T8-9 complex by a short, narrow ridge; T8 directly posterior of T5; T9 a rounded buccal projection of T8, distinguished by a shallow cleft on the anterior slope of the loph; anterior cingulum weakly present on T1, continuing to poorly defined lingual cingulum between bases of T1 and T4; buccal cingulum weakly expressed and appears to be present between the bases of T3 and T6; posterior cingulum appears absent, but may have been obliterated by interstitial masticatory wear; M^1^ has three roots (large anterior, elongate lingual, and postero-buccal).

Remarks.

The holotype is referred to *Leggadina* based on the extreme development of the accessory cusp, diamond-shaped T1 with supplementary cusp, and posteriorly-displaced T1 and T4. The size of this species (M^1^ dimensions: 3.52 × 1.86 mm) is enough to easily distinguish it from all other species of *Leggadina* (Fig. 6). In comparison, *L. macrodonta* has a largest-recorded M^1^ length of 3.22 mm and M^1^ width of 1.75 mm (QMF57276 and QMF57267, respectively. Measurements from Klinkhamer & Godthelp (2015). The holotype and only specimen of *L. irvini* is worn, so the full length of the M^1^ is likely greater than measured in Fig. 6. Reliance on size differences alone to inform taxonomic decisions is sometimes problematic (e.g. Price, 2008b), but here we note additional morphologies that allow separation of *L. irvini* from the largest previously known species within the genus, *L. macrodonta*. In addition to overall size, the holotype of *L. irvini* is further distinguished from all known specimens of *L. macrodonta* by having a more robust accessory cusp and T1 on M^1^. Some species of *Leggadina* display minor variation in these cusps, however, the degree of expression of these features in *L. irvini* can only be assessed further following recovery of additional specimens.

*L. irvini* is further distinguished from *Leggadina webbi* sp. nov (see below) and *L. gregoriensis* by its larger accessory cusp.

**Figure 7:**
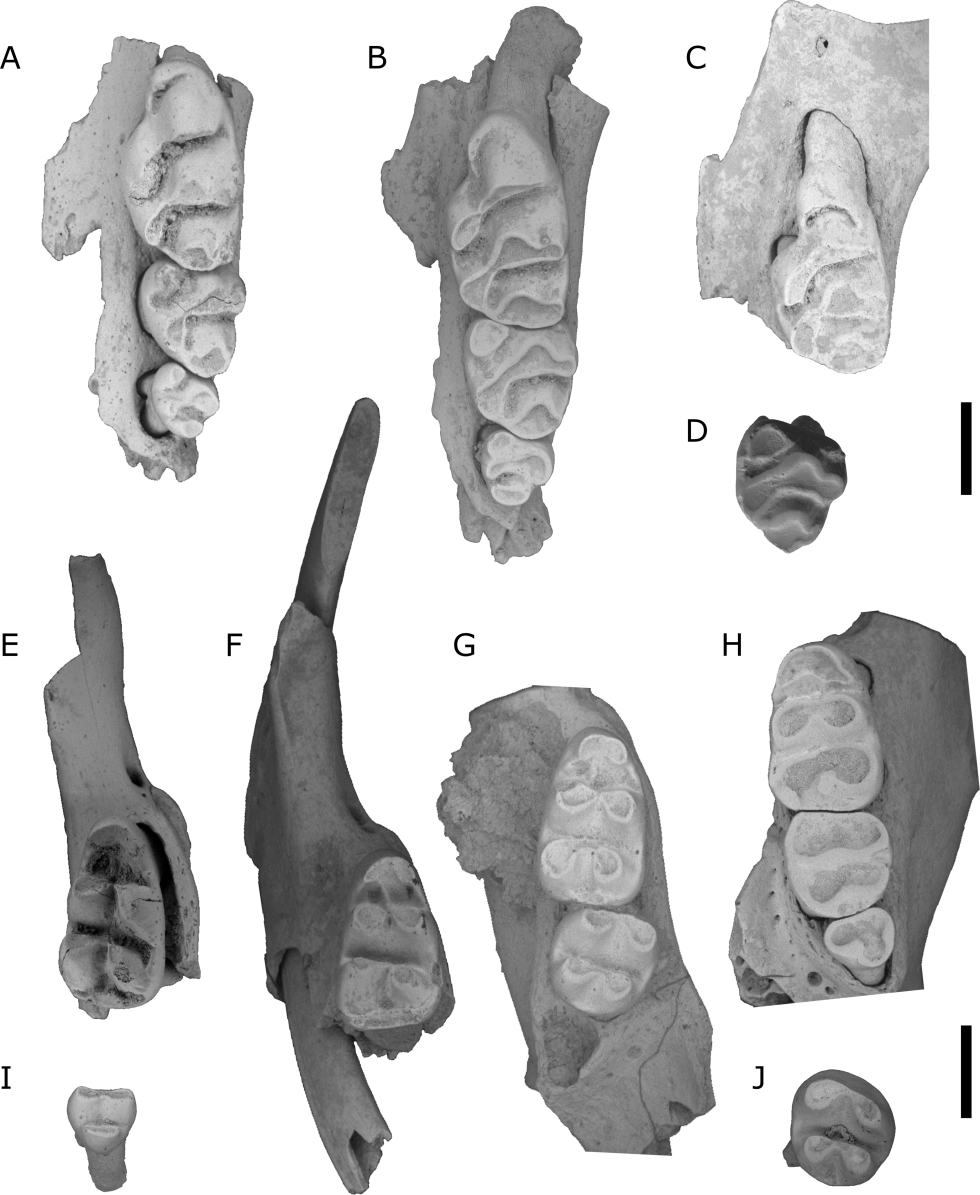
*Leggadina webbi* sp. nov. (A) QMF55629 left maxilla; (B) QMF55628 left maxilla; (C) QMF55635 left maxilla fragment with M^1^; (D) QMF55636 left M^2^; (E) QMF55631 right mandible fragment with M_1_; (F) QMF55640 right mandible fragment with I_1_ and M_1_; (G) QMF55641 right mandible with M_1−2_; (H) QMF55638 left mandible; (I) QMF55645 right M_3_, removed from mandible; (J) QMF55639 left M_2_. Scale bar = 1 mm.

***Leggadina*** ***webbi*** sp. nov. (Fig. 7, Appendix S1)

Holotype. QMF55628 left maxilla with M^1−3^.

Type locality. QML1313; Speaking Tube Cave System, Mount Etna, eastern central Queensland.

Other material. QML1313; Speaking Tube Cave System, Mount Etna: QMF55629 left maxilla, QMF55630 left M^1^, QMF55631 right mandible fragment with M_1_, QMF55632 right mandible, QMF55633 left mandible, QMF55634 left mandible.QML1311H; Speaking Tube Cave System, Mount Etna, QMF55642 left M^1^, QMF55634 left M^1^, QMF55644 left M^1^, QMF55645 right mandible; QML1311J; Speaking Tube Cave System, Mount Etna: QMF55635 left maxilla fragment with M^1^, QMF55636 left M^2^, QMF55637 left mandible, QMF55638 left mandible, QMF55639 left M_2_, QMF55640 right mandible fragment with I_1_ and M_1_, QMF55641 right mandible;

Etymology. This species is named for Gregory Webb in recognition of his support for studies of cave fossils in Queensland, and his broader contributions to Australian palaeontology.

Diagnosis. A species of *Leggadina* that contains the following unique combination of characters: M^1^/_1_ slightly longer than half of molar row (Appendix S1); M^3^/_3_ moderately reduced in size (proportionally smaller than that of *Pseudomys* spp., but less-reduced than *L. forresti* and *L. lakedownensis*); accessory cusp on M^1^ relatively small (smaller than that of L. *forresti* and *L. lakedownensis*); T1 on M^1^ not greatly enlarged; upper molars moderately overlapping; anterior palatal foramina posteriorly narrow.

Description.

Maxilla. No complete maxillae are known. The anterior palatal foramina are posteriorly narrow.

M^1^. Crown elongate and oval-shaped in occlusal outline, with marked indent on antero-lingual edge; most specimens have a relatively small (for a species of *Leggadina*) accessory cusp; accessory cusp commonly confined to lingual margin on base of T2 (one specimen, QMF55635, has it wrapping around the anterior of T2); T1 accessory cusp absent; T1 oval or diamond-shaped in occlusal outline; T1 postero-lingual of T2, joining after mastication-related wear to T1 bis; T1 bis variably developed, positioned immediately lingual of T2; T2 similar in size to T1; T3 reduced in size; T4 directly posterior of T1; T5 directly posterior of T2 and antero-buccal of T4; T4 crescent-shaped in occlusal outline, joined to T5 by a narrow ridge; T4 joined to T8 by a low ridge, more evident after moderate to heavy wear; T5 triangular in occlusal outline, slightly larger than T4; T6 oval to sub-circular in shape, directly buccal of T5 when unworn, reduced but less so than T3; T7 absent; T8 rounded, sloping posteriorly; T9 reduced and fused to T8, still distinct when unworn; M^1^ has three roots (large anterior, elongate lingual, and small postero-buccal).

M^2^. T1 well-developed, oval to semicircular-shaped in occlusal outline; T2 absent; T3 variably expressed, small if present (e.g., QMF55628, 55635); T1 in contact with postero-lingual margin of T8 of M^1^; T1 directly anterior to T4; T4 tear-shaped, semicircular in occlusal outline after wear; T4 postero-lingual of T5, joined by narrow ridge; T5 triangular in occlusal outline, pointing anteriorly; T6 not heavily reduced, fused to buccal corner of T5; T4 joined at base to T8 by low ridge; T8 triangular in occlusal outline; T9 heavily reduced, not discernible on some specimens; M^2^ has three roots (antero-buccal, lingual, and postero-buccal).

M^3^. Reduced, but no hyper-reduced specimens known; crown tapers posteriorly to approximately half total width in occlusal outline; T1 in contact with postero-lingual margin of T8 of M^2^; T1 sub-circular to semicircular in occlusal outline; T1 directly anterior of T4 and antero-lingual of T5-6 complex; T4 small, tear-shaped, joined to T5-6 complex by narrow ridge; T5-6 complex rounded, tapering to junction with T4; T6 indistinguishable from T5, possibly absent; T8-9 complex reduced, narrower than T4-6 loph; M^3^ has one major root that is bifurcated.

M_1_. Crown elongate with relatively large gaps between lophs; anterior loph commonly relatively narrow (in comparison to other species of *Leggadina*), boomerang or semicircular-shaped in occlusal outline (depending on degree of wear); antero-lingual and antero-buccal cusps fused; antero-lingual cusp slopes to base of metaconid; antero-lingual cusp has second, smaller ridge sloping to buccal margin of base of metaconid; antero-buccal cusp slopes to base of protoconid; protoconid directly posterior of antero-buccal cusp and immediately buccal of metaconid; protoconid and metaconid subequal in size, both tear-shaped, pointed towards junction at midpoint of anterior margin of second loph; protoconid and metaconid merge after heavy wear, but still distinguishable by indents at midpoint of anterior and posterior margins of loph; hypoconid directly posterior of protoconid and immediately buccal of entoconid; hypoconid joined to protoconid by a low ridge on the buccal margin of the crown, almost forming a buccal cingulid; entoconid slightly larger than hypoconid; hypoconid and entoconid tear-shaped in occlusal outline, tapering to point slightly anterior of anterior margins of both cusps; short and broad posterior cingulid cusp on well-developed posterior cingulid; posterior cingulid commonly wraps around base of hypoconid, continuing to base of protoconid; M_1_ has two roots (anterior and posterior).

M_2_. Roughly square-shaped in occlusal outline; two lophs comprising the protoconid-metaconid and hypoconid-entoconid; protoconid-metaconid loph broader than hypoconid-entoconid loph, and more robust at base; all major cusps tear-shaped in occlusal outline; protoconid tapers antero-lingually to meet tapering point of metaconid at midpoint of anterior margin of anterior loph; hypoconid joins entoconid in likewise fashion; low shelf or (variably) small posterior cingulid cusp on posterior margin of crown between bases of hypoconid and entoconid; M_2_ has two roots (anterior and posterior).

M_3_. Very small; all known specimens have two lophs; protoconid and metaconid elliptical in occlusal outline, aligned with lophs of other molars; posterior loph approximately same size and shape as protoconid; M_3_ has one root, bifurcated into anterior and posterior.

Remarks. The upper dentition of *L. webbi* is similar to that of some of the very small species of *Pseudomys*. However, we assign such material to *Leggadina* on the basis of its large posterior palatal foramina and broad posterior cingulid cuspid on M_1_. Additionally, *L. webbi* differs from *L. forresti* by having less overlap between the upper molars and having a proportionally smaller T1 and accessory cusp on M^1^. *L. webbi* differs from *L. lakedownensis* by having less overlap between the upper molars; having a proportionally smaller M^1^/_1_ and larger M^3^/_3_, proportionally smaller T1 and accessory cusp on M^1^, and having posteriorly narrow anterior palatal foramina. *L. webbi* differs from *L. macrodonta* by being smaller, lacking a T1 supplementary cusp (commonly present on *L. macrodonta*), having a more developed anterior medial ridge and more developed buccal cingulid on the M_1_.

*L. webbi* shares several features with *L. gregoriensis* including size, possession of a relatively small accessory cusp on M^1^, and a relatively small T1 on the M^1^. It differs by lacking a defined T7 on M^1^.

**Figure 8:**
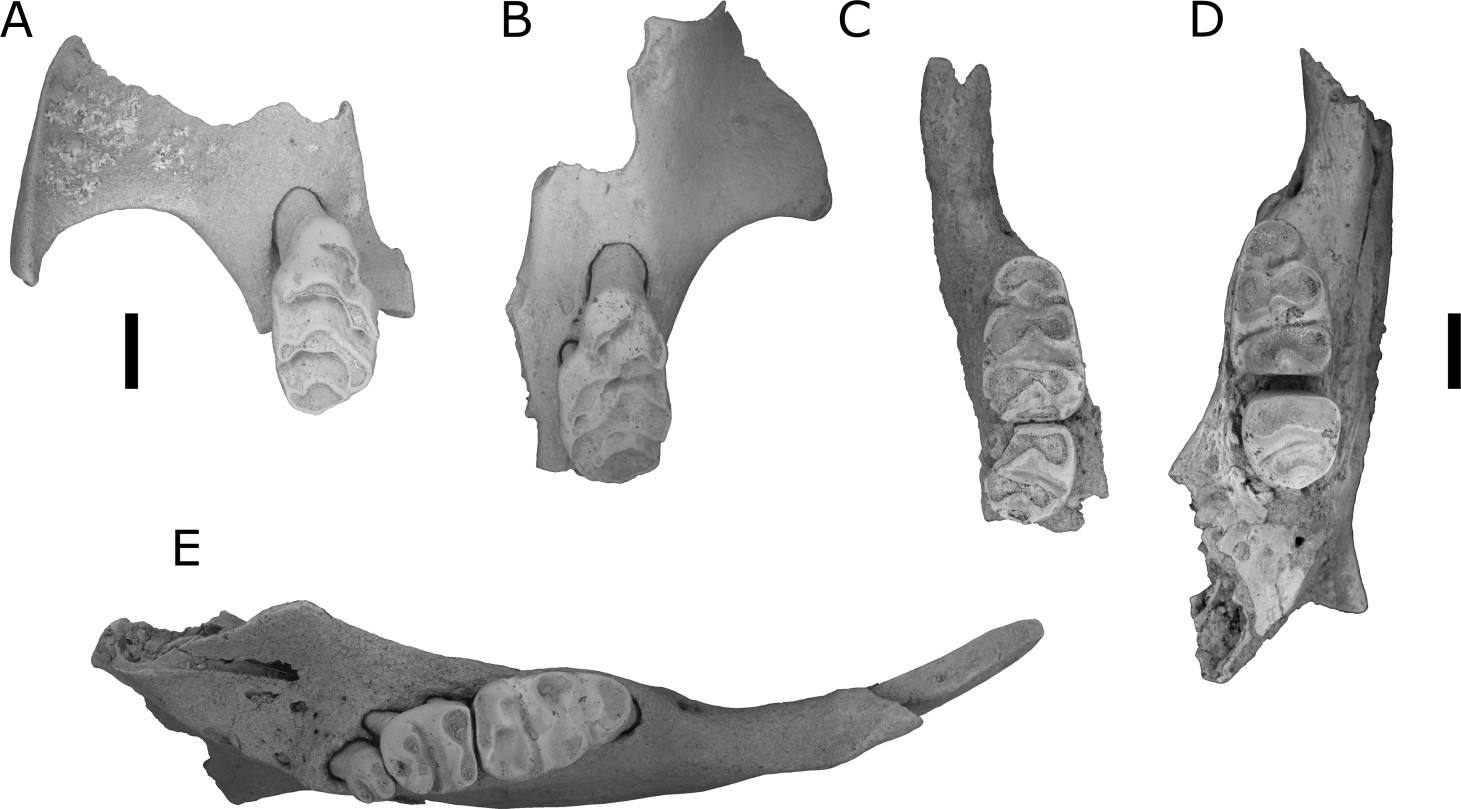
L. macrodonta. (A) QMF55622 right maxilla fragment with M^1^; (B) QMF55623 left maxilla fragment with M^1^; (C) QMF55626 partial right mandible; (D) QMF 55627 left mandible; (E) QMF55625 left mandible. Scale bars = 1 mm.

***Leggadina macrodonta*** Klinkhamer & Godthelp (2015) (Figs. 5 and 8)

Locality: All specimens are from the site 5C and adjacent Woodbine Creek, both on the western bank of the Leichardt River, Floraville Station, northwest Queensland.

Diagnosis: See Klinkhamer & Godthelp (2015) for some diagnostic characters for the maxilla and association dentition. Here we add the following characters: a moderately large-toothed (Fig. 6, Table 1) species of *Leggadina* with a posteriorly rounded anterior palatal foramen, and a relatively narrow anterior loph on M_1_.

**Table 1:** Dental dimensions of additional specimens of *L. macrodonta*. All measurements in millimetres.

	*N*	mean	sd	min	max
M^1^ length	3	2.92	0.03	2.86	2.96
M^1^ width	3	1.61	0.02	1.58	1.65
M_1_ length	3	2.31	0.03	2.26	2.34
M_1_ width	3	1.47	0.01	1.45	1.48
M_2_ length	3	1.39	0.04	1.32	1.47
M_2_ width	2	1.39	0.08	1.31	1.46
M_3_ length	1	0.73	na	na	na
M_3_ width	1	0.78	na	na	na
M_1−3_ length	1	4.42	na	na	na

Referred specimens: QMF55622 partial right maxilla with M^1^; QMF55623 partial left maxilla with M^1^; QMF55624 right maxilla fragment with M^1^; QMF55625 left mandible with I_1_, M_1−3_; QMF55626 partial right mandible with M_1−2_; QMF55627 left mandible with M_1−2_.

Description.

Additional features of the maxilla: The anterior palatal foramen ends in line with the anterior margin of the lingual root of M^1^; buccal margin of anterior palatal foramen at least partially curved, but all specimens are too damaged to determine the shape of the posterior end of the foramen.

Dentary. All known lower molars exhibit moderate to heavy wear. One specimen (QMF55625) preserves the I_1_, but no specimens have an intact coronoid process, articular facet or angular process.

M_1_. Roughly ovoid in occlusal outline, but with more rectangular posterior end; first loph narrower than second or third lophs; first loph rounded, kidney-shaped in occlusal outline; antero-buccal and antero-lingual cuspids cannot be distinguished on any available specimens due to heavy wear; weak buccal ridge sloping from antero-buccal cuspid to base of protoconid, but lacking on antero-lingual cuspid; antero-lingual cusp with very small ridge sloping to buccal margin of base of metaconid; protoconid posterior and slightly buccal of antero-buccal cuspid; metaconid directly lingual of protoconid; protoconid and metaconid subequal in size, each tapering towards the other, joined by tall ridge; hypoconid directly posterior of protoconid and directly buccal of entoconid; hypoconid and entoconid subequal in size, slightly smaller than protoconid and metaconid; hypoconid and entoconid both tear-shaped, each tapering towards the other, meeting at anterior margin of loph; low ridge connects base of protoconid to base of hypoconid, but does not continue into a buccal cingulid on postero-buccal corner of hypoconid; posterior cingulid cuspid well developed, lenticular in occlusal outline, tapering buccally along base of posterior face of hypoconid; two roots present (anterior and posterior).

M_2_. Almost square-shaped in occlusal outline; protoconid-metaconid loph forms broadest part of crown; protoconid larger than metaconid, becoming less obvious after heavy wear; protoconid and metaconid both tear-shaped, although protoconid is longer; protoconid and metaconid meet on anterior margin of loph (and crown) at midline of molar row; hypoconid-entoconid loph slightly narrower than protoconid-metaconid loph; hypoconid posterior and slightly lingual of protoconid; entoconid lingual and slightly anterior of hypoconid; hypoconid and entoconid both tear-shaped, each tapering toward the other to meet at the midpoint between them; small posterior cingulid cuspid nestled immediately posterior of junction between hypoconid and entoconid; two roots present (anterior and posterior).

M_3_. Only one specimen (QMF55625) preserves the M_3_; crown sub-circular in occlusal outline; protoconid and metaconid fused, occlusal surface peanut-shaped; posterior loph small, directly posterior of metaconid; appears to have only one root, but may bifurcate within the dentary.

Remarks.

Klinkhamer & Godthelp (2015) did not describe the lower dentition of *L. macrodonta* stating that no specimens from the type locality could be definitively assigned to the species. However, from our assessment of the available, and some new, fossil specimens of mandibles and lower molars from the type locality, we consider that such material is unequivocally *Leggadina*. Furthermore, considering that this species is morphologically distinct from all known species of *Leggadina*, and only a single morphospecies of *Leggadina* has been identified in the deposit, we refer the new and previously undescribed lower dentition to *L. macrodonta*. Additional specimens have also been recovered from another deposit (Woodbine Creek) a short distance from the type locality. Although *L. macrodonta* was named for its bulky M^1^ and was considered to be the largest-known member of the genus at the time of publication (Klinkhamer & Godthelp, 2015), *L. irvini* sp. nov. is substantially larger (Figs. 5 and 6).

## Discussion

### Dental and functional morphology in *Leggadina*

The enlarged accessory cusp on the M^1^ is probably the most striking feature of the dentition in most species of *Leggadina*. It is much less-developed in the oldest-known species (*L. gregoriensis*), suggesting that its larger size in most other species of *Leggadina* may be a derived trait. The degree of wear on many specimens suggests that the accessory cusp is highly functional, effectively forming an anterior-most fourth loph on the M^1^. The two extant species, *L. forresti* and *L. lakedownensis*, commonly exhibit a median constriction of the accessory cusp, effectively dividing it in two. This feature is also observed in *L. macrodonta* and *L. irvini.* Although its function is unclear it may be that the constriction creates a narrower cutting edge, a useful tool for processing invertebrate prey.

### Phylogenetic relationships

Determining the precise phylogenetic position of species within *Leggadina* is difficult due to the fragmentary nature of the known fossil record of skeletal and dental material. It is also hampered by a poor understanding of morphological diversity in cranial and dental characters among Australian murids generally. However, we assume that the oldest known species, *L. gregoriensis* from the early Pleistocene, is likely to possess more ancestral traits than species found in geologically younger deposits. Three distinctive features of *Leggadina* are less-developed in *L. gregoriensis* than other species within the genus: the development of the accessory cusp on M^1^; the enlargement of M^1^ relative to M^3^, and the development of the T1 supplementary cusp on M^1^. We interpret the condition of these features in *L. gregoriensis* as the plesiomorphic state for the genus.

*L. webbi* retains several characters that appear to be plesiomorphic within the genus. These include the enlargement of the accessory cusp and T1 on M^1^, and the reduction of M^3^/_3_. This association of characters may represent an evolutionary grade, as they are also seen in the oldest known species, *L. gregoriensis*. While *L. gregoriensis*, as the oldest known species of the genus, is unsurprisingly plesiomorphic, *L. webbi* is much younger and was likely contemporaneous with species that are still extant today. We therefore suggest that *L. webbi* represents a plesiomorphic species of *Leggadina* and based on its interpreted palaeoecology likely represents a ‘relic’ lineage from the earlier diversification of the genus. ‘Relic’ or ‘Ghost’ lineages are known from the Middle Pleistocene Mt. Etna fauna including plesiomorphic bandicoots, possums, koalas, and marsupial ‘lions’ (Hocknull, 2005; Hocknull, 2009; Hocknull et al., 2007; Price & Hocknull, 2011). We hypothesise that the rodent fauna reflects an early radiation of murids into closed rainforest environments and subsequent extinction in these environments during the late Middle Pleistocene and Late Pleistocene. Similar diversification and biased mesic extinction has been suggested within the dasyurid marsupials from Mt. Etna also (Cramb, Hocknull & Webb, 2009; Cramb & Hocknull, 2010b).

Is it possible that *L. webbi* represents something close to the actual ancestor of the genus? Klinkhamer & Godthelp (2015) suggested that *Leggadina* evolved from a rainforest-inhabiting ancestor, and indeed the most basal extant taxa in the Australo-Papuan murine radiation occur in New Guinean rainforests (Rowe et al., 2008). However, based on molecular evidence the genus *Leggadina* is nested within a clade whose extant members (*Pseudomys*, *Notomys*, *Mastacomys*, and *Zyzomys*) are not currently found in tropical rainforest habitats (Rowe et al., 2008). If, then, *L. webbi* represents the ancestral condition of *Leggadina* both morphologically and ecologically it suggests that *Leggadina* diversified within mesic (rainforest) habitats first, with subsequent secondary xeric-adaptation of its derived end member species and subsequent extinction of the mesic-adapted plesiomorphic forms.

Alternatively, it may indicate that the predominately xeric-adapted Australian murid clades have all experienced mesic radiations sometime in the past and these lineages are now missing due to the overriding extinction bias of these habitats throughout the Quaternary due to intensifying aridity (e.g., Hocknull et al., 2007; Price, 2012b).

*L. irvini* is the least-known species of the genus due to a lack of fossil specimens. On the basis of gross morphology, the relatively large accessory cusp and T1 on the M^1^ appear to be derived, indicating that it is probably closer to the crown species of *Leggadina* than to *L. gregoriensis*. The overall large size of *L. irvini* could possibly indicate a morphocline derived from the smallest species through *L. macrodonta* to *L. irvini*. Apart from *L. gregoriensis* and *L. webbi*, the remaining members of the genus all share a similar level of development of characters such as the enlargement of the accessory cusp and T1 on M^1^, and the presence of a T1 supplementary cusp on M^1^. This precludes the construction of a clearer evolutionary narrative for the end members of this genus.

**Figure 9:**
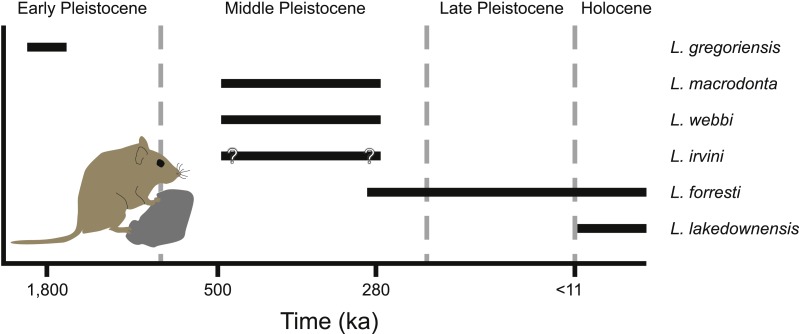
Temporal distribution of species of *Leggadina*.

### Spatial and temporal occurrence

*Leggadina* has a long history in northern Australia with the oldest described species of the genus, *L. gregoriensis*, occurring in deposits dating to the Early Pleistocene (Fig. 9; Klinkhamer & Godthelp, 2015). A lack of well-dated fossil deposits between 1.8–0.5 Ma limits our understanding of the radiation within the genus. By the Middle Pleistocene, at least three species of *Leggadina* occur, namely *L. webbi* and *L. forresti* from the Mt. Etna region, and *L. macrodonta* from Floraville. An additional species, *L. irvini* from Chillagoe, is interpreted as probably being of middle Pleistocene age. The fate of *L. macrodonta* is unclear as it is so far known from only two deposits, however, *L. webbi* appears to have persisted until ca. 280 ka.

A paucity of radiometric dating limits our understanding of the timing of the origin of species such as *L. lakedownensis* and *L. irvini*. Both taxa lack firm geochronological control, but were present by the Middle-Late Pleistocene. The presence of *L. lakedownensis* within fossil deposits of the Chillagoe region is not surprising, as the type locality of the species (Lakeland Downs) is relatively close (<150 km to the North-East) and extant populations occur in the region today (Moro & Kutt, 2008). Interestingly though, the absence of *L. lakedownensis* from older Middle –Late Pleistocene deposits in the Chillagoe area may indicate that it is a geologically recent arrival in the area. *L. irvini* is something of a puzzle. Only a single specimen is known from a site of uncertain age. Why it was replaced by the smaller *L. lakedownensis* in the Chillagoe area cannot be ascertained without better understanding of its age and contemporary faunas.

*Leggadina webbi* is a common element of fossil assemblages from the Mt. Etna region that are dominated by rainforest-adapted taxa (Hocknull, 2005; Hocknull et al., 2007). Its loss from the region is likely associated with intensifying aridity that began during the mid-Bruhnes climatic event that saw a major faunal turnover at Mt. Etna whereby rainforest-adapted fauna were replaced by extant species that presently live in xeric habitats (Hocknull et al., 2007; Price, 2012b). Significantly though, this faunal turnover event brought *L. forresti* to the area. Indeed, fossils from Mt. Etna itself are the oldest record (ca. 280 ka) for this species and can therefore be utilised as a calibration point in future phylogenetic assessments using molecular clocks. *L. forresti* survived locally until at least the late Holocene, being recorded from deposits such as the Pleisto-Holocene Colosseum Chamber (Price et al., 2015). We are currently unaware of any extant records of *L. forresti* in the Mt. Etna region, but the local small-bodied extant mammal fauna is likely to be incompletely known with additional records of rodents that may either still be present in the region or very recently extinct (Cramb & Hocknull, 2010a). For example, the most common small-bodied dasyurid marsupial found within the Colosseum Chamber deposit is the Stripe-faced Dunnart, *Sminthopsis macroura* (Cramb, Hocknull & Webb, 2009), a species that was only found extant in the area as recently as 2007 (represented by specimen QMJM18420). It is thus possible that extant populations of *L. forresti* may yet be discovered in the area.

### A cautionary note regarding palaeoecology

Extant species of *Leggadina* are omnivores that dig simple burrows (Moro & Kutt, 2008; Reid, 2008). They are found in a variety of habitats ranging from deserts to tropical woodlands (Kutt & Kemp, 2005). The discovery of *L. webbi* in fossil deposits interpreted as representing closed rainforest palaeoenvironments indicates *Leggadina* had an even broader habitat range in the past. This may be taken as a cautionary note for any researcher attempting to reconstruct past environments on the basis of the presumed habitat preferences of single fossil taxa using only extant relatives. Palaeoecological reconstructions should ideally be based on multiple taxa with congruent habitat preferences, with consideration of anatomical specialisations, ecological guilds and relevant palaeoclimatic data.

For example, extant Tree Kangaroos (*Dendrolagus* spp.) are commonly associated with rainforest habitats, yet fossil Tree Kangaroos (*Bohra* spp.) evidently had broader ecological tolerances, being found in assemblages interpreted as woodlands (Dawson, 2004; Prideaux & Warburton, 2009) as well as rainforests (Hocknull, 2005). Similarly, recently extant Bilbies (*Macrotis* spp.) are exclusively found in xeric habitats, yet fossil Bilbies (*Liyamayi dayi*) inhabited rainforests (Travouillon et al., 2014).

It must be remembered that the long span of geological time will include many habitats hosting lineage associations that have no analogue today (Lundelius Jr, 1989).

Interestingly, *Leggadina* is part of the ‘*Pseudomys* division’ of hydromine rodents, a group which is notably absent from extant rainforest communities. In contrast, middle Pleistocene deposits at Mount Etna (e.g., QML 1311H) contain six species of *Pseudomys* division murines. At least five of these appear to have been endemic to rainforest habitats, as they are only found in deposits with other more typical rainforest-inhabiting taxa. This suggests that modern assemblages of rainforest murines are not a good analogue for those of the middle Pleistocene.

## Conclusions

The two new species of *Leggadina* described in this paper, *L. irvini* and *L. webbi*, demonstrate that the genus once displayed greater diversity of morphology and ecology. Moreover, with six species of *Leggadina* now recognised–four extinct and two extant–it is clear that species diversity within the genus was greater in the past. While the extinction of *L. webbi* appears to be tied to climate-change driven habitat loss, the causes for the wider decline of the genus in recent times is unclear. It is, however, a pattern also apparent in other Australian mammal groups such as koalas (Price, 2008a; Black et al., 2014; Price, 2012a). This present study adds to the growing list of small-bodied mammal extinctions that occurred in Australia, alongside the extinction of the megafauna, throughout the late Quaternary. It demonstrates that the Quaternary extinctions included representatives from many of the most successful extant lineages, such as murids, peramelids, dasyurids and phascolarctids. We contend that determining the chronological, palaeoecological and palaeoclimatic context for the extinction of these small-bodied fauna is of equal importance to that of the megafauna extinction debate.

The fossil record of Australasian rodents has received relatively little research attention, but recent work by Klinkhamer & Godthelp (2015), coupled with this study, has greatly enlarged our understanding of at least one genus. The task must now be to extend this work to the 35 other murine genera known from Australo-Papua.

##  Supplemental Information

10.7717/peerj.5639/supp-1 Appendix S1 Molar measurements of *Leggadina webbi* sp. nov All measurements in millimetres.

## Institutional abbreviations

QMF: Queensland Museum fossil specimen
QMJM: Queensland Museum modern specimen
QML: Queensland Museum fossil locality

## Additional Information and Declarations

### Data Availability

The following information was supplied regarding data availability: The molar measurements of Leggadina webbi sp. nov are provided in Appendix S1.

### New Species Registration

The following information was supplied regarding the registration of a newly described species: ***Publication LSID:*** urn:lsid:zoobank.org:pub:3C34C73B-4800-4256-BC9E-0E7B50689024 **Leggadina irvini LSID:** urn:lsid:zoobank.org:act:06906882-7329-45DD-BCC8-C3474DC925BE, **Leggadina webbi LSID:** urn:lsid:zoobank.org:act:E420DF0C-C7BD-421A-B7A2-7E3EDC598588.
