# Supplementary material for: Short-tailed mice with a long fossil record: the genus Leggadina (Rodentia: Muridae) from the Quaternary of Queensland, Australia
Source: PeerJ. 2018 Sep 21;6:e5639. doi: 10.7717/peerj.5639 (PMC6152458; doi:10.7717/peerj.5639)
Supplement: Appendix S1 — All measurements in millimetres. [file peerj-06-5639-s001.docx]

Appendix 1. Molar measurements of *Leggadina webbi* sp. nov. All measurements in millimetres.

| M^1^ Length |  |  |  |  |  |  |  | M^1^ Width | |  |  |  |  |  |
| --- | --- | --- | --- | --- | --- | --- | --- | --- | --- | --- | --- | --- | --- | --- |
|  | N | Mean | SD | Min | Max | CV |  |  | N | Mean | SD | Min | Max | CV |
| 1311 H |  |  |  |  |  |  |  |  | 1 | 1.47 | na | na | na |  |
| 1311 J | 8 | 2.47 | 0.15 | 2.14 | 2.62 |  |  |  | 8 | 1.45 | 0.05 | 1.37 | 1.54 |  |
| 1313 | 7 | 2.58 | 0.11 | 2.43 | 2.70 |  |  |  | 9 | 1.48 | 0.06 | 1.41 | 1.58 |  |
| All | 15 | 2.52 | 0.14 | 2.14 | 2.70 | 5.63 |  |  | 18 | 1.46 | 0.06 | 1.37 | 1.58 | 3.79 |
|  |  |  |  |  |  |  |  |  |  |  |  |  |  |  |
| M^2^ Length |  |  |  |  |  |  |  | M^2^ Width | |  |  |  |  |  |
|  | N | Mean | SD | Min | Max | CV |  |  | N | Mean | SD | Min | Max | CV |
| 1311 J | 1 | 1.37 | na | na | na |  |  |  | 1 | 1.29 | na | na | na |  |
| 1313 | 3 | 1.44 | 0.03 | 1.42 | 1.47 |  |  |  | 3 | 1.20 | 0.04 | 1.17 | 1.25 |  |
| All | 4 | 1.43 | 0.04 | 1.37 | 1.47 |  |  |  | 4 | 1.23 | 0.06 | 1.17 | 1.29 |  |
|  |  |  |  |  |  |  |  |  |  |  |  |  |  |  |
| M^3^ Length |  |  |  |  |  |  |  | M^3^ Width | |  |  |  |  |  |
|  | N | Mean | SD | Min | Max | CV |  |  | N | Mean | SD | Min | Max | CV |
| 1313 | 1 | 0.86 | na | na | na |  |  |  | 1 | 0.81 | na | na | na |  |
|  |  |  |  |  |  |  |  |  |  |  |  |  |  |  |
| M^1-3^ Length | |  |  |  |  |  |  | M_1-3_ Length | |  |  |  |  |  |
|  | N | Mean | SD | Min | Max | CV |  |  | N | Mean | SD | Min | Max | CV |
| 1311 J |  |  |  |  |  |  |  |  | 2 | 3.99 | 0.02 | 3.98 | 4.01 |  |
| 1313 | 1 | 4.31 | na | na | na |  |  |  |  |  |  |  |  |  |
|  |  |  |  |  |  |  |  |  |  |  |  |  |  |  |
| M_1_ Length | |  |  |  |  |  |  | M_1_ Width | |  |  |  |  |  |
|  | N | Mean | SD | Min | Max | CV |  |  | N | Mean | SD | Min | Max | CV |
| 1311 H | 3 | 2.05 | 0.15 | 1.88 | 2.18 |  |  |  | 3 | 1.25 | 0.04 | 1.21 | 1.28 |  |
| 1311 J | 15 | 1.97 | 0.05 | 1.88 | 2.06 | 2.41 |  |  | 16 | 1.26 | 0.05 | 1.14 | 1.32 | 4.37 |
| 1313 | 13 | 2.03 | 0.07 | 1.93 | 2.15 | 3.26 |  |  | 13 | 1.29 | 0.06 | 1.22 | 1.42 | 4.32 |
| All | 31 | 2.00 | 0.07 | 1.88 | 2.18 | 3.62 |  |  | 32 | 1.27 | 0.06 | 1.14 | 1.42 | 4.36 |
|  |  |  |  |  |  |  |  |  |  |  |  |  |  |  |
| M_2_ Length | |  |  |  |  |  |  | M_2_ Width | |  |  |  |  |  |
|  | N | Mean | SD | Min | Max | CV |  |  | N | Mean | SD | Min | Max | CV |
| 1311 H | 1 | 1.24 | na | na | na |  |  |  | 1 | 1.24 | na | na | na |  |
| 1311 J | 4 | 1.24 | 0.04 | 1.2 | 1.3 |  |  |  | 4 | 1.22 | 0.02 | 1.19 | 1.24 |  |
| 1313 | 9 | 1.36 | 0.12 | 1.23 | 1.63 |  |  |  | 9 | 1.30 | 0.08 | 1.24 | 1.49 |  |
| All | 14 | 1.32 | 0.12 | 1.20 | 1.63 | 8.76 |  |  | 14 | 1.27 | 0.07 | 1.19 | 1.49 | 5.79 |
|  |  |  |  |  |  |  |  |  |  |  |  |  |  |  |
| M_3_ Length | |  |  |  |  |  |  | M_3_ Width | |  |  |  |  |  |
|  | N | Mean | SD | Min | Max | CV |  |  | N | Mean | SD | Min | Max | CV |
| 1311 H | 1 | 0.74 | na | na | na |  |  |  | 1 | 0.80 | na | na | na |  |
| 1311 J | 2 | 0.77 | 0.13 | 0.68 | 0.86 |  |  |  | 2 | 0.84 | 0.00 | 0.84 | 0.84 |  |
| All | 3 | 0.76 | 0.09 | 0.68 | 0.86 |  |  |  | 3 | 0.83 | 0.02 | 0.80 | 0.84 |  |

| M^1^ Length |  |  |  |  |  |  |  | M^1^ Width | |  |  |  |  |  |
| --- | --- | --- | --- | --- | --- | --- | --- | --- | --- | --- | --- | --- | --- | --- |
|  | N | Mean | SD | Min | Max | CV |  |  | N | Mean | SD | Min | Max | CV |
| 1311 H |  |  |  |  |  |  |  |  | 1 | 1.47 | na | na | na |  |
| 1311 J | 8 | 2.47 | 0.15 | 2.14 | 2.62 |  |  |  | 8 | 1.45 | 0.05 | 1.37 | 1.54 |  |
| 1313 | 7 | 2.58 | 0.11 | 2.43 | 2.70 |  |  |  | 9 | 1.48 | 0.06 | 1.41 | 1.58 |  |
| All | 15 | 2.52 | 0.14 | 2.14 | 2.70 | 5.63 |  |  | 18 | 1.46 | 0.06 | 1.37 | 1.58 | 3.79 |
|  |  |  |  |  |  |  |  |  |  |  |  |  |  |  |
| M^2^ Length |  |  |  |  |  |  |  | M^2^ Width | |  |  |  |  |  |
|  | N | Mean | SD | Min | Max | CV |  |  | N | Mean | SD | Min | Max | CV |
| 1311 J | 1 | 1.37 | na | na | na |  |  |  | 1 | 1.29 | na | na | na |  |
| 1313 | 3 | 1.44 | 0.03 | 1.42 | 1.47 |  |  |  | 3 | 1.20 | 0.04 | 1.17 | 1.25 |  |
| All | 4 | 1.43 | 0.04 | 1.37 | 1.47 |  |  |  | 4 | 1.23 | 0.06 | 1.17 | 1.29 |  |
|  |  |  |  |  |  |  |  |  |  |  |  |  |  |  |
| M^3^ Length |  |  |  |  |  |  |  | M^3^ Width | |  |  |  |  |  |
|  | N | Mean | SD | Min | Max | CV |  |  | N | Mean | SD | Min | Max | CV |
| 1313 | 1 | 0.86 | na | na | na |  |  |  | 1 | 0.81 | na | na | na |  |
|  |  |  |  |  |  |  |  |  |  |  |  |  |  |  |
| M^1-3^ Length | |  |  |  |  |  |  | M_1-3_ Length | |  |  |  |  |  |
|  | N | Mean | SD | Min | Max | CV |  |  | N | Mean | SD | Min | Max | CV |
| 1311 J |  |  |  |  |  |  |  |  | 2 | 3.99 | 0.02 | 3.98 | 4.01 |  |
| 1313 | 1 | 4.31 | na | na | na |  |  |  |  |  |  |  |  |  |
|  |  |  |  |  |  |  |  |  |  |  |  |  |  |  |
| M_1_ Length | |  |  |  |  |  |  | M_1_ Width | |  |  |  |  |  |
|  | N | Mean | SD | Min | Max | CV |  |  | N | Mean | SD | Min | Max | CV |
| 1311 H | 3 | 2.05 | 0.15 | 1.88 | 2.18 |  |  |  | 3 | 1.25 | 0.04 | 1.21 | 1.28 |  |
| 1311 J | 15 | 1.97 | 0.05 | 1.88 | 2.06 | 2.41 |  |  | 16 | 1.26 | 0.05 | 1.14 | 1.32 | 4.37 |
| 1313 | 13 | 2.03 | 0.07 | 1.93 | 2.15 | 3.26 |  |  | 13 | 1.29 | 0.06 | 1.22 | 1.42 | 4.32 |
| All | 31 | 2.00 | 0.07 | 1.88 | 2.18 | 3.62 |  |  | 32 | 1.27 | 0.06 | 1.14 | 1.42 | 4.36 |
|  |  |  |  |  |  |  |  |  |  |  |  |  |  |  |
| M_2_ Length | |  |  |  |  |  |  | M_2_ Width | |  |  |  |  |  |
|  | N | Mean | SD | Min | Max | CV |  |  | N | Mean | SD | Min | Max | CV |
| 1311 H | 1 | 1.24 | na | na | na |  |  |  | 1 | 1.24 | na | na | na |  |
| 1311 J | 4 | 1.24 | 0.04 | 1.2 | 1.3 |  |  |  | 4 | 1.22 | 0.02 | 1.19 | 1.24 |  |
| 1313 | 9 | 1.36 | 0.12 | 1.23 | 1.63 |  |  |  | 9 | 1.30 | 0.08 | 1.24 | 1.49 |  |
| All | 14 | 1.32 | 0.12 | 1.20 | 1.63 | 8.76 |  |  | 14 | 1.27 | 0.07 | 1.19 | 1.49 | 5.79 |
|  |  |  |  |  |  |  |  |  |  |  |  |  |  |  |
| M_3_ Length | |  |  |  |  |  |  | M_3_ Width | |  |  |  |  |  |
|  | N | Mean | SD | Min | Max | CV |  |  | N | Mean | SD | Min | Max | CV |
| 1311 H | 1 | 0.74 | na | na | na |  |  |  | 1 | 0.80 | na | na | na |  |
| 1311 J | 2 | 0.77 | 0.13 | 0.68 | 0.86 |  |  |  | 2 | 0.84 | 0.00 | 0.84 | 0.84 |  |
| All | 3 | 0.76 | 0.09 | 0.68 | 0.86 |  |  |  | 3 | 0.83 | 0.02 | 0.80 | 0.84 |  |
